# Translation and cross-cultural adaptation of the *Yale Pharyngeal Residue Severity Rating Scale* into Brazilian Portuguese

**DOI:** 10.1016/j.bjorl.2024.101470

**Published:** 2024-07-15

**Authors:** Roberta Seabra Venite, Leandro Pernambuco, Roberta Gonçalves da Silva, Suely Mayumi Motonaga Onofri

**Affiliations:** a Universidade Estadual Paulista (Unesp), Faculdade de Filosofia e Ciências, Marília, SP, Brazil; b Universidade Federal da Paraíba (UFPB), João Pessoa, PB, Brazil

**Keywords:** Deglutition, Endoscopy, Translation, Cross-cultural comparison, Back-translation

## Abstract

**Objective:**

To report the process of translation and cross-cultural adaptation of the*Yale Pharyngeal Residue Severity Rating Scale* into Brazilian Portuguese.

**Methods:**

Methodological study approved by the Ethics Committee of the Institution (nº 5.166.256). The English original scale was translated into Brazilian Portuguese following suggested in the literature guidelines and recommendations after authorization from the authors of the original instrument, and involved the following reported steps of (1) Translation, (2) Synthesis of translations, (3) Determination of the applicability of the translated version 4) Back-translation, (5) Synthesis of the back-translated versions, and (6) Final synthesis. The translations and back-translations were performed by two bilingual translators. The research committee constituted three specialists who considered whether the linguistic, semantics, conceptual, idiomatic, and contextual equivalence of the translations and back-translations were. In Step 3, the raters consisted of three Speech-Language Pathologists and five ENT physicians divided into two subgroups (less than 5 years of professional expertise, and more than 5 years of professional expertise).

**Results:**

Step 1 was carried out properly by the translators, in Step 2 the translated version was prepared after minor adjustments. In Step 3, the raters reported that they found no difficulties in applying the scale. The Cronbach's Alpha coefficient was 0.995, demonstrating high internal consistency of the instrument, and the analysis of the Intraclass Correlation Coefficient (ICC) among the eight raters was 0.994 with a confidence interval between 0.990 and 0.998, demonstrating excellent agreement, regardless of experience. The research committee judged the last to be adequate and not to require adjustments.

**Conclusion:**

The Brazilian Portuguese version of the*Yale Pharyngeal Residue Severity Rating Scale* is presented in this study.

This is a methodological study – No level of evidence.

## Highlights

• This study determined the translation of the Yale Pharyngeal Scale to Portuguese.
• This study describes guidelines for translation and cross-cultural adaptation.
• The application of this new instrument to clinics and researchers is ready.

## Introduction

Fiberoptic Endoscopic Evaluation of Swallowing (FEES) is an established method that permits a direct view of the pharynx, larynx anatomical structures, and pharyngeal phase of deglutition.^1^ Some protocols describe findings such as premature oral spillage, pharyngeal residues, penetration, and aspiration in dysphagic populations.^2^ Usually, the pharyngeal residue scales are scored by the valleculae and/or pyriform sinuses site, and the amount of residue in these regions.^2^, ^3^ The FEES seems to be superior to videofluoroscopy mainly in visualizing pharynx residue, and it is associated with aspiration risk, has a substantial influence on the safety of swallowing, and can lower quality of life.^4^, ^5^, ^6^

Therefore, attributing pharyngeal residue is essential for supporting the medical report, enabling adequate therapeutic planning for each individual with oropharyngeal dysphagia, and making recommendations for safe oral alimentation.^7^ Many different scales for pharyngeal residue severity rating involve scoring residue site, amount, and clearing management of pooled bolus, but few scales have been developed and validated that are, easily applied clinically which is a desirable feature of such an instrument.^8^, ^9^, ^10^ *The Yale Pharyngeal Residue Rating Scale* (YPRSRS) is an anatomically defined and image-based tool that includes, a 5-point ordinal scale with a separate residue severity score for the valleculae and the pyriform sinuses thus displaying all the desirable features.^11^

There has been an increase in the translation and cross-cultural adaptation of instruments into Brazilian Portuguese due to the lack of validated instruments for oropharyngeal dysphagia; past practice saw the application of particular institutional instruments based on previous research, but without validation. The key to resolving this issue does not involve the development of new scales, which is usually expensive and time-consuming, and often results in instruments that cannot be readily compared with previous scales. The YPRSRS was chosen due to its high validity and reliability; it has been translated into and validated in German, Turkish, and Italian, and has proved to be easy to use by professionals regardless of experience.^12^, ^13^, ^14^

An instrument to be utilized in Brazil must be translated according to guidelines that result in semantic, content, and concept equivalence with the original model, and for later validation while giving due consideration to cultural diversity.^15^, ^16^ There is no consensus among researchers on methodologies for translation and cross-cultural adaptation; there are numerous variations, and detailed information about this process is usually not provided.^17^ The validation of many research instruments has treated this step as unimportant, which compromises the reliability and validity of the translated instrument. This study aimed to describe the process of translation and cross-cultural adaptation of the YPRSRS into Brazilian Portuguese.

## Methods

This is a methodological study restricted to translation and cross-cultural adaptation. It was approved by the Human Research Ethics Committee of the Institution under number 5.166.256. All participants agreed to participate and signed the free and informed consent form. The YPRSRS is a residue severity score scale for the valleculae and the pyriform sinuses comprising a 5-point ordinal scale from none to severe: none (0%), trace (1%–5%), mild (5%–25%), moderate (25%–50%) and severe (>50%).

We followed the guidelines and recommendations suggested in the literature for translation and cross-cultural adaptation from English into Brazilian Portuguese.^15^ The process began after we received authorization from the authors of the original instrument it involved the following steps described further below: (1) Translation, (2) Synthesis of translations, (3) Determination of the applicability of the translated version, (4) Back-translation, (5) Synthesis of the back-translated versions, and (6) Final synthesis.

1) In the translation step, two qualified translators who are speech therapists, native in the target language and fluent in English language, independently translated the instrument with due consideration for conceptual, semantic, idiomatic, contextual, and linguistic equivalence while avoiding literal translation.
2) Subsequently, the translation was synthesized through the consensus of three native speakers of the target language, two speech-language pathologists, and one ENT physician, who were enrolled as a research committee (Committee 1) and were experienced specialists in dysphagia. A single version was constructed by comparing translation versions (Version 1) and evaluating any semantic, idiomatic, conceptual, linguistic, and contextual discrepancies.
3) After the synthesis of the translations, the applicability of the translated Version 1 was verified. For this step, we formed a new group (Committee 2) consisting of a speech-language pathologist, a head and neck surgeon, and an ENT. The judges completed a questionnaire about choosing the most suitable translation and provided comments related to feasibility, layout, difficulties, and suggestions. After receiving the questionnaire and analyzing the answers by Committee 1 based on this consensus, a new version in Brazilian Portuguese (Version 2) was finalized. This Version 2 was judged by raters (Committee 3) who included three speech-language pathologists and five ENTs who were divided into two subgroups: one with more than five years of experience, and the other with less than five years. This Version 2 was applied in a natural context to analyze the application of the items regarding structure and suitability, yielding evidence of validity based on the response. The cognitive questionnaire was sent by email and consisted of an explanatory video using the randomized images from the original study: two images from each of the five levels selected from the original scale. The raters could analyze the images, make contributions regarding operational difficulties, and verify the applicability of this instrument.
4) This new version of Brazilian Portuguese, Version 2, was sent for back-translation to two bilingual translators, who were North American, native speakers of English, and fluent in Brazilian Portuguese. The translators have not had previous contact with the original version and were unaware of the instrument.
5) For the synthesis of the back-translated versions, both back-translation versions were analyzed and compared by Committee 1 with due consideration of conceptual, semantic, idiomatic, contextual, and linguistic equivalence. The adjustments were decided by consensus and a synthesis version was generated (Version 3).
6) In the final synthesis, Committee 1 compared the original and final versions in the target language (Version 2) in terms of semantic, idiomatic, conceptual, linguistic, experiential, and contextual equivalences.

The Cronbach's alpha coefficient was used to measure internal consistency and the Intraclass Correlation Coefficient (ICC) agreement between raters. The analyses were carried out with IBM SPSS v26.0® software, and the significance level was set at 5%.

## Results

Two translations of the original YPRSRS instrument in English were independently created and a consensus Version 1 was built by Committee 1 with minor adaptations. We did not observe conceptual, semantic, idiomatic, contextual, or linguistic discrepancies between the two Brazilian Portuguese versions.

To the applicability of the translated version 1, Committee 2 suggested the following points: (a) “Up wall to a quarter full”, and “Up wall to half full” are terms whose interpretation is subjective; (b) There is no difference between uni and bilateral content, and (c) The term “epiglottic ligament” should be changed to “glossoepiglottic ligament”. Committee 1 by consensus accepted only the third suggestion respecting the original version of the scale and changed it to “glossoepiglottic ligament” according to anatomical nomenclature. Still in Step 3, after the considerations of Committee 2, the new Brazilian Portuguese (Version 2) (Table 1) was finished by Committee 1.

**Table 1:** The Brazilian Portuguese YPRSRS (Version 2).

Definições para gravidade de resíduo na valécula			Definições para gravidade do resíduo nos recessos piriformes		
I	Nenhum	0% Sem resíduo	I	Nenhum	0% Sem resíduo
II	Vestígio	1%–5% Fina camada revestindo a mucosa	II	Vestígio	1%–5% Fina camada revestindo a mucosa
III	Leve	5%–25% Ligamento da epiglote visível	III	Leve	5%–25% Preenchido até um quarto
IV	Moderado	25%–50% Ligamento da epiglote coberto	IV	Moderado	25%–50%Preenchido até a metade
V	Grave	>50% Preenchido até a borda da epiglote	V	Grave	>50% Preenchido até a prega ariepiglótica

Committee 3 formed by three speech-language pathologists, and five ENT physicians had no difficulties in applying Version 2 to analyze 20 random images from the original validation study. Cronbach's Alpha had a value of 0.995, demonstrating high internal consistency of the instrument, and the analysis of the Intraclass Correlation Coefficient (ICC) among the eight judges was 0.994 with a confidence interval between 0.990 and 0.998, demonstrating an excellent agreement between judges.

The back-translation of Version 2 was performed by two native English speakers, who received the instrument by e-mail. In the next step, Committee 1 created a synthesis of the back-translated versions after comparing them. We did not observe conceptual, semantic, idiomatic, contextual, or linguistic discrepancies between the two back-translated versions.

Finally, in the last step, Committee 1 compared and analyzed the original and final versions in the target language, and judged the Brazilian Portuguese version of the YPRSRS (Table 2) adequate in terms of semantic, idiomatic, conceptual, linguistic, experiential, and contextual equivalences.

**Table 2:** The Final Brazilian Portuguese YPRSRS version.

Resíduos em valéculas			Resíduos em seios piriformes		
I	Nenhum	0% Sem resíduo	I	Nenhum	0% Sem resíduo
II	Vestígio	1%–5% Fina camada revestindo a mucosa	II	Vestígio	1%–5% Fina camada revestindo a mucosa
III	Leve	5%–25% Ligamento glossoepiglótico visível	III	Leve	5%–25% Preenchido até um quarto
IV	Moderado	25%–50% Ligamento glossoepiglótico coberto	IV	Moderado	25%–50% Preenchido até a metade
V	Grave	>50% Preenchido até a borda da epiglote	V	Grave	>50% Preenchido até a prega ariepiglótica

## Discussion

The YPRSRS is an anatomically defined, image-based assessment of post-swallow pharyngeal residue severity based on residue location (vallecula and pyriform sinus) and amount (none, trace, mild, moderate, and severe) as observed during FEES.^11^ This study determined the cultural equivalences and linguistic aspects of YPRSRS based on the translation and adaptation of this instrument for Brazilian Portuguese, in preparation for the next steps of the validation process.

As the pharyngeal residue can have deleterious consequences, since 1996 there have been different scales for the analysis of pharyngeal residue.^8^, ^9^, ^10^, ^11^ The FEES evaluation has more sensitivity to pharyngeal residue than videofluoroscopy.^2^, ^4^ The YPRSRS has been shown to have high validity and reliability in studies in German, Turkish, and Italian.^12^, ^13^, ^14^

Recently the YPRSRS was translated and validated, but as different guidelines and recommendations for translation and cross-cultural adaptation were followed, the most important issue is to resolve discrepancies between cultures and languages. The process for producing the German version was based on guidelines with six stages, for translation and back-translation.^18^ The content analysis by experts interviewed showed an excellent overall understanding of the pre-final version and high relevance for German-speaking populations. The development of the Turkish version involved translation and back-translation and the final version was sent to the original authors.^17^, ^19^ Finally, the Italian version involved forward translation and its review for consensus, and backward translations and their review for consensus by specialists in dysphagia.^17^ We thus observed different methods for translation and cross-cultural adaptation, and studies describe strategies and stages of this process.^15^, ^16^, ^17^, ^18^, ^19^

The tests of reliability and construct validity in the original and translated instruments showed excellent results.^12^, ^13^, ^14^ No differences were found between the groups with different levels of professional experience. Cronbach's alpha of our Brazilian Portuguese version presented values of 0.9, indicating high internal consistency of the instrument, and the Intraclass Correlation Coefficient (ICC) between the eight judges was 0.994, indicating a high level of interrater agreement. The selected raters had different levels of experience grouped as more than five years and less than five years, and included three speech-language pathologists and five ENTs. This result confirms that the instrument is a practical tool that does not require high levels of FEES expertise.

Other validated residue scales^9^, ^20^ are time-consuming to administer and impractical in the daily clinical practice of professionals; thus, our scale holds great promise for applicability in the research setting. This new version will contribute to the development of scales with clinical applicability and enhance research on oropharyngeal dysphagia through an instrument rigorously translated and cross-culturally adapted to Brazilian Portuguese.^15^

A significant limitation of this study is the applicability of images from the original validation study, with high-definition quality, unlike in Brazil, is unclear, but we believe that this difference would not change the results. The application of this new instrument should be of great value to clinics and researchers on oropharyngeal dysphagia.

## Conclusion

We translated and cross-culturally adapted the YPRSRS into Brazilian Portuguese, following guidelines and recommendations suggested in the literature, and judged by specialists to be appropriate for use in clinical and research settings, and is thus ready for the next steps of the validation process.

## Conflicts of interest

The authors declare no conflicts of interest.

## References

[1] Langmore S.E., Kenneth S.M.A., Olsen N. (1988). Fiberoptic endoscopic examination of swallowing safety: a new procedure. Dysphagia.

[2] Langmore S.E. (2017). History of fiberoptic endoscopic evaluation of swallowing for evaluation and management of pharyngeal dysphagia: changes over the years. Dysphagia..

[3] Murray J., Langmore S.E., Ginsberg S., Dostie A. (1996). The significance of accumulated oropharyngeal secretions and swallowing frequency in predicting aspiration. Dysphagia..

[4] Labeit B., Ahring S., Boehmer M. (2022). Comparison of simultaneous swallowing endoscopy and videofluoroscopy in neurogenic dysphagia. J Am Med Dir Assoc..

[5] Butler S.G., Markley L., Sanders B., Stuart A. (2015). Reliability of the penetration aspiration scale with flexible endoscopic evaluation of swallowing. Ann Otol Rhinol Laryngol..

[6] Pisegna J.M., Kaneoka A., Leonard R., Langmore S.E. (2018). Rethinking residue: determining the perceptual continuum of residue on FEES to enable better measurement. Dysphagia..

[7] Schindler A., Baijens L.W.J., Geneid A., Pizzorni N. (2022). Phoniatricians and otorhinolaryngologists approaching oropharyngeal dysphagia: an update on FEES. Eur Arch Otorhinolaryngol..

[8] Neubauer P.D., Hersey D.P., Leder S.B. (2016). Pharyngeal residue severity rating scales based on fiberoptic endoscopic evaluation of swallowing: a systematic review. Dysphagia..

[9] Kaneoka A.S., Langmore S.E., Krisciunas G.P., Field K., Scheel R., McNally E. (2013). The Boston residue and clearance scale: preliminary reliability and validity testing. Folia Phoniatr Logop..

[10] Farneti D. (2008). Pooling score: an endoscopic model for evaluating severity of dysphagia. Acta Otorhinolaryngol Ital..

[11] Neubauer P.D., Rademaker A.W., Leder S.B. (2015). The Yale pharyngeal residue severity rating scale: an anatomically defined and image-based tool. Dysphagia..

[12] Gerschke M., Schöttker-Königer T., Förster A., Netzebandt J.F., Beushausen U.M. (2019). Validation of the german version of the Yale pharyngeal residue severity rating scale. Dysphagia..

[13] Atar Y., Atar S., Ilgin C., Anarat M.E.A., Uygan U., Uyar Y. (2022). Validity and reliability of the turkish translation of the Yale pharyngeal residue severity rating scale. Dysphagia..

[14] Nordio S., Maistrello L., D’Imperio D. (2023). Validity and reliability of the italian translation of the yale pharyngeal residue severity rating scale. Acta Otorhinolaryngol Ital..

[15] Pernambuco L., Espelt A., Magalhães H.V., Lima K.C. (2017). Recommendations for elaboration, transcultural adaptation and validation process of tests in Speech, Hearing and Language Pathology. Codas..

[16] Beaton D.E., Bombardier C., Guillemin F., Ferraz M.B. (2000). Guidelines for the process of cross-cultural adaptation of self-report measures. Spine..

[17] Sousa V.D., Rojjanasrirat W. (2011). Translation, adaptation and validation of instruments or scales for use in cross-cultural health care research: a clear and user-friendly guideline. J Eval Clin Pract..

[18] Schmitt M., Eid M. (2007). Guidelines for the translation of foreign-language measurement instruments. Diagnostica..

[19] Gjersing L., Caplehorn J.R., Clausen T. (2010). Cross-cultural adaptation of research instruments: language, setting, time and statistical considerations. BMC Med Res Methodol..

[20] Curtis J.A., Borders J.C., Perry S.E., Dakin A.E., Seikaly Z.N., Troche M.S. (2022). Visual analysis of swallowing efficiency and safety (VASES): a standardized approach to rating pharyngeal residue, penetration, and aspiration during Fees. Dysphagia..

